# Measurement and monitoring of anions, cations and metals in landfill leachate in Iranian metropolises

**DOI:** 10.1016/j.dib.2018.11.013

**Published:** 2018-11-06

**Authors:** Nadali Alavi, Akbar Eslami, Mohammad Hossien Saghi

**Affiliations:** a Environmental and Occupational Hazards Control Research Center, Shahid Beheshti University of Medical Sciences, Tehran, Iran; b Department of Environmental Health Engineering, Sabzevar University of Medical Sciences, Sabzevar, Iran

**Keywords:** Selected Iranian metropolises, Landfill leachates, Heavy metals

## Abstract

Leachate generation is a major problem for municipal solid waste (MSW) landfills and causes significant threat to surface and groundwater. Samples were collected from the landfills of Iranian metropolises (Ahwaz, Mashhad, Tehran (before and after treatment plant), Isfahan, Tabriz, Hamedan, Rasht, Sanandaj and Qazvin) based on the standard sampling methods and transferred to the laboratory. Cr, Cd, Hg, Pb, Ni, As, Ca, Mg, Fe, Mn, Na, K, Zn, Al, Ba, Li, Sr, Ti and V were analyzed. The pH values for the ten leachate samples obtained from 4.57 to 8.95. The results showed the amount of some metals in Iranian landfill sites higher than the DOE standards for agricultural irrigation and surface water.

**Specifications table**

**Table:** 

**Subject area**	Environmental Engineering
**More specific subject area**	Landfill leachate monitoring
**Type of data**	Statistical data
**How data was acquired**	Samples were collected from the landfills of Iranian metropolises based on standard sampling methods and transferred to the laboratory and kept at 4 °C The amount of Cr, Cd, Hg, Pb, Ni, As, Ca, Mg, Fe, Mn, Na, K, Zn, Al, Ba, Li, Sr, Ti and V were detected with ICP-MS.
**Data format**	Table
**Experimental factor**	Monitoring of trace and heavy metals in landfill leachate
**Experimental features**	Determine the concentration levels of trace and heavy metals.
**Data source location**	Research Center for Occupational and Environmental Hazardous Factors, Shahid Beheshti University of Medical Sciences, Tehran, Iran
**Data accessibility**	Data are presented in the article

**Value of data**

• Based on the data, the amount of some metals in landfill leachate in Iranian landfill sites higher than the DOE standards for agricultural irrigation and surface water.
• Obtained data were used for assessment of soil, surface and ground water pollutions around the landfill sites.
• The results can be useful for wide application on leachate treatment plant.

## 1 Data

**Fig. 1:**
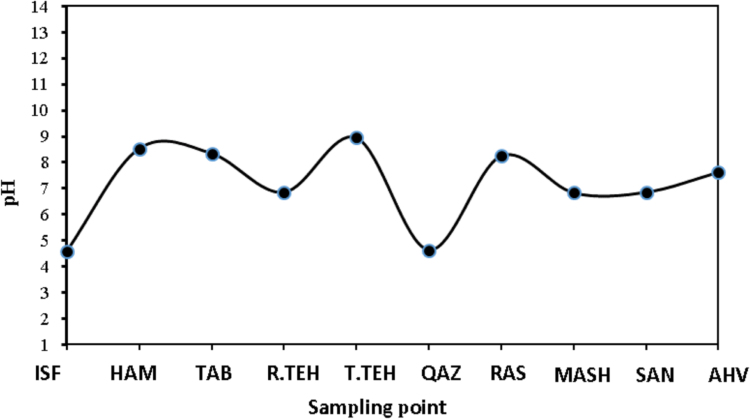
The amount of pH value in landfill leachate sites.

The data obtained from the pH analysis of leachates in ten sites are summarized in Fig. 1. The pH values for the ten leachate samples examined ranged from 4.57 to 8.95, with mean values of 7.143 ± 1.45. The value of trace and heavy metals characteristics of landfill leachate sites are presented in (Table 1, Table 2, Table 3). Samples were collected from the landfills of Iranian metropolises (Ahwaz, Mashhad, Tehran (before and after leachate treatment plant), Isfahan, Tabriz, Hamedan, Rasht, Sanandaj and Qazvin based on standard sampling methods and transferred to the laboratory). In this sites *anions, cations,* metals and heavy metals (Cr, Cd, Hg, Pb, Ni, As, Ca, Mg, Fe, Mn, Na, K, Zn, Al, Ba, Li, Sr, Ti, V) were analyzed [1]. The results showed the amount of some metals in landfill leachate in Iranian landfill sites higher than the DOE standards for agricultural irrigation and surface water.

**Table 1:** Amount of trace and heavy metals in landfills leachate of Iranian metropolises (First sampling).

Parameters	City												
	Isfahan	Hamedan	Tabriz	Raw Teh.	Treated Teh.	Qazvin	Rasht	Mashhad	Sanandaj	Ahvaz	Max	Min	S.D
Li (ppb)	27.97	52.50	31.39	30.67	26.16	21.47	14.07	20.93	49.80	58.46	58.46	14.07	14.24
Na (ppm)	20	123	47	83	55	35	28	51	152	304	303.80	19.67	81.95
Mg(ppm)	3.9	3.7	2.0	8.9	2.4	4.4	1.7	8.1	7.4	29.1	29.12	1.67	7.71
Al (ppb)	60	11	12	7	4	42	9	7	4	14	60.46	3.90	17.79
K (ppm)	17	120	34	24	13	16	21	34	29	43	120.48	13.45	29.81
Ca (ppm)	17	8	4	15	4	19	7	38	19	61	60.92	3.90	16.80
Ti (ppb)	0.9	14.1	10.1	4.2	0.3	5.1	4.0	1.5	1.4	2.5	14.08	0.26	4.22
V (ppb)	7.4	2.1	4.3	2.1	4.5	3.6	2.7	7.8	8.4	8.4	8.37	2.13	2.47
Cr (ppb)	3.6	7.9	15.6	6.0	4.6	3.3	2.5	5.9	5.0	5.7	15.64	2.50	3.54
Mn (ppb)	181.2	52.0	8.1	55.6	35.9	122.2	33.9	90.3	38.5	86.9	181.15	8.07	48.58
Fe (ppb)	67.6	67.4	36.6	61.5	15.1	1768.5	308.3	159.5	58.4	272.8	1768.52	15.06	504.74
Ni (ppb)	<5	13.5	3.2	5.0	5.2	3.3	6.3	10.2	7.6	10.2	13.54	3.24	3.32
Cu (ppb)	14.2	4.6	3.3	3.7	4.4	8.6	7.5	11.0	14.3	8.3	14.33	3.25	3.92
Zn (ppb)	37.1	10.9	5.6	6.1	3.5	38.7	17.3	19.2	19.9	21.4	38.68	3.50	11.70
As (ppb)	2.4	10.5	5.0	5.3	3.5	3.65	4.50	3.56	3.66	3.69	10.46	2.35	2.12
Sr (ppb)	152	87	55	195	57	105	46	174	140	403	402.97	45.52	100.25
Cd (ppb)	<1	<1	<1	<1	<1	<1	<1	<1	<1	0.01	0.01	0.01	0.00
Ba (ppb)	5.7	4.2	2.6	7.1	2.2	8.1	3.4	7.3	6.8	9.4	9.41	2.24	2.34
Hg (ppb)	<1	<1	<1	<1	2.64	<1	<1	2.25	<1	<1	2.64	2.25	0.20
Pb (ppb)	3.15	2.98	3.98	2.50	3.50	5.50	3.65	1.98	3.50	3.59	5.50	1.98	0.89

**Table 2:** Amount of trace and heavy metals in landfills leachate of Iranian metropolises (Second sampling).

Parameters	City												
	Isfahan	Hamedan	Tabriz	Raw Teh.	Treated Teh.	Qazvin	Rasht	Mashhad	Sanandaj	Ahvaz	Max	Min	S.D
Li (ppb)	25.20	52.00	31.39	31.00	26.16	21.47	14.07	20.93	49.80	68.23	68.23	14.07	16.20
Na (ppm)	22.00	123.00	40.00	83.27	35.00	35.17	15.00	50.62	151.74	336.00	336.00	15.00	92.62
Mg(ppm)	3.94	3.50	2.01	9.90	2.60	4.39	1.80	8.13	7.40	29.80	29.80	1.80	7.93
Al (ppb)	60.46	10.89	11.73	6.94	3.90	41.77	9.23	7.23	4.42	13.94	60.46	3.90	17.79
K (ppm)	16.92	120.48	33.50	24.33	13.45	15.58	21.04	34.30	29.14	43.01	120.48	13.45	29.81
Ca (ppm)	18.00	9.00	3.90	28.00	8.00	19.23	7.50	37.93	19.31	60.92	60.92	3.90	16.53
Ti (ppb)	0.98	14.08	10.13	4.19	0.26	5.13	3.97	1.46	1.45	2.49	14.08	0.26	4.21
V (ppb)	7.45	2.13	4.25	2.13	4.50	3.56	2.65	7.81	8.37	8.35	8.37	2.13	2.47
Cr (ppb)	3.55	7.87	15.64	6.01	4.55	3.25	2.50	9.20	5.00	5.67	15.64	2.50	3.67
Mn (ppb)	181.15	52.00	8.07	55.64	35.90	122.24	33.85	90.33	38.55	86.89	181.15	8.07	48.58
Fe (ppb)	67.64	67.42	36.56	61.49	15.06	1768.52	334.00	165.30	58.41	272.76	1768.52	15.06	504.80
Ni (ppb)	5.00	13.54	3.24	5.02	5.23	3.25	6.30	10.22	7.63	10.24	13.54	3.24	3.22
Cu (ppb)	14.17	4.63	3.25	3.69	4.35	8.59	7.51	10.98	14.33	8.34	14.33	3.25	3.92
Zn (ppb)	38.00	10.91	5.56	6.06	3.50	38.68	17.30	19.20	19.94	21.40	38.68	3.50	11.84
As (ppb)	2.35	10.46	5.00	5.33	3.50	3.65	4.50	3.56	3.66	3.69	10.46	2.35	2.12
Sr (ppb)	152.19	87.31	54.80	194.93	57.16	105.26	45.52	174.38	140.47	402.97	402.97	45.52	100.25
Cd (ppb)	<1	<1	<1	1.00	<1	<1	<1	<1	<1	0.01	1.00	0.01	0.50
Ba (ppb)	5.66	4.22	2.59	9.00	2.24	8.11	3.35	7.30	6.79	9.41	9.41	2.24	2.52
Hg (ppb)	<1	<1	1.00	<1	2.64	1.00	<1	2.25	<1	1.00	2.64	1.00	0.72
Pb (ppb)	3.15	2.98	4.50	3.00	3.50	5.50	3.65	1.98	3.50	3.56	5.50	1.98	0.89

**Table 3:** Amount of trace and heavy metals in landfills leachate of Iranian metropolises (Third sampling).

Parameters	**Sample code**												
	Isfahan	Hamedan	Tabriz	Raw Teh.	Treated Teh.	Qazvin	Rasht	Mashhad	Sanandaj	Ahvaz	Max	Min	S.D
Li (ppb)	25.20	52.00	31.39	31.00	26.16	21.47	16.35	20.93	49.80	83.50	83.50	16.35	19.46
Na (ppm)	22.00	123.00	40.00	83.27	35.00	35.17	22.00	50.62	151.74	382.00	382.00	22.00	104.57
Mg(ppm)	3.94	3.50	2.01	9.90	2.60	4.39	1.79	8.13	7.40	36.00	36.00	1.79	9.70
Al (ppb)	60.46	10.89	13.65	6.94	3.90	41.77	9.23	6.55	4.42	13.94	60.46	3.90	17.78
K (ppm)	19.85	120.48	33.50	22.00	13.45	16.58	21.04	34.30	33.65	43.01	120.48	13.45	29.61
Ca (ppm)	18.00	9.00	3.90	32.00	8.00	19.23	8.60	37.93	19.31	60.92	60.92	3.90	16.65
Ti (ppb)	0.98	14.08	10.13	6.00	0.22	5.13	3.97	1.46	1.45	2.49	14.08	0.22	4.24
V (ppb)	7.45	2.13	4.25	2.13	5.60	3.56	2.65	7.81	8.37	8.35	8.37	2.13	2.47
Cr (ppb)	4.55	7.87	15.64	6.01	6.50	3.25	2.50	9.20	5.00	5.67	15.64	2.50	3.55
Mn (ppb)	181.15	52.00	8.07	55.64	45.00	122.24	33.85	90.33	38.55	86.89	181.15	8.07	48.01
Fe (ppb)	67.64	67.42	36.56	61.49	15.06	1800.20	453.20	165.30	58.41	332.50	1800.20	15.06	516.42
Ni (ppb)	5.00	13.54	3.24	5.02	5.23	3.25	6.30	10.22	7.63	10.24	13.54	3.24	3.22
Cu (ppb)	14.17	4.63	3.25	3.69	4.35	8.59	7.51	10.98	14.33	8.34	14.33	3.25	3.92
Zn (ppb)	38.00	10.91	5.56	6.06	3.50	38.68	17.30	19.20	19.94	21.40	38.68	3.50	11.84
As (ppb)	3.36	10.46	5.00	5.33	3.33	3.65	4.50	3.56	3.66	3.69	10.46	3.33	2.05
Sr (ppb)	152.19	87.31	54.80	316.00	57.16	105.26	45.52	186.32	140.47	402.97	402.97	45.52	112.81
Cd (ppb)	<1	<1	1.00	1.00	<1	0.89	<1	<1	<1	0.01	1.00	0.01	0.42
Ba (ppb)	5.66	4.22	2.59	9.00	2.24	8.11	3.35	7.30	6.79	9.41	9.41	2.24	2.52
Hg (ppb)	<1	0.90	1.00	<1	2.25	1.00	<1	2.25	<1	1.00	2.25	0.90	0.60
Pb (ppb)	3.15	3.98	4.50	4.23	3.50	6.50	3.65	1.98	3.50	3.56	6.50	1.98	1.09

## 2 Experimental design, materials and methods

The leachate samples were prepared with the coordination of the waste management organization of Iranian metropolises from different cities. The criterion for selecting cities was determined with the geographic location of these cities in Iran, having a landfill, having leachate drainage. Also, the selected city must have an acceptable population and should be the provincial capital. The basis on this criteria, ten point selected of Iran (Isfahan, Hamedan, Tabriz, Tehran (before treatment, after treatment), Qazvin, Rasht, Mashhad, Sanandaj, and Ahvaz) [2], [3]. Samples were collected from the landfills of Iranian metropolises based on standard sampling methods and transferred to the laboratory and kept at 4 °C. Grab samples (1 L) were collected glass bottles, preserved with 1 g of sodium azide and returned via overnight courier to the laboratory in coolers containing ice-blocks [4]. Samples were filtered through a 0.45 µm glass fiber filters before storage at 4 °C until extraction [2], [5]. The amount of Cr, Cd, Hg, Pb, Ni, As, Ca, Mg, Fe, Mn, Na, K, Zn, Al, Ba, Li, Sr, Ti and V were detected with ICP-MS.
