# Supplementary material for: Measurement and monitoring of anions, cations and metals in landfill leachate in Iranian metropolises
Source: Data Brief. 2018 Nov 6;21:1818–22. doi: 10.1016/j.dib.2018.11.013 (PMC6260328; doi:10.1016/j.dib.2018.11.013)
Supplement: Supplementary file 1 — Supplementary material [file mmc1.doc]

Conflict of Interest and Authorship Conformation Form

Please check the following as appropriate:

- All authors have participated in (a) conception and design, or analysis and interpretation of the data; (b) drafting the article or revising it critically for important intellectual content; and (c) approval of the final version.
- This manuscript has not been submitted to, nor is under review at, another journal or other publishing venue.
- The authors have no affiliation with any organization with a direct or indirect financial interest in the subject matter discussed in the manuscript
- The following authors have affiliations with organizations with direct or indirect financial interest in the subject matter discussed in the manuscript:

Author’s name Affiliation

Nadali Alavi Research Center for Occupational and Environmental hazardous Factors, Shahid Beheshti University of Medical Sciences, Tehran, Iran,

Akbar Eslami Research Center for Occupational and Environmental hazardous Factors, Shahid Beheshti University of Medical Sciences, Tehran, Iran

Mohammad Hossien Saghi Department of Environmental Health Engineering, Sabzevar University of Medical Sciences, Sabzevar, Iran, Gmail: saghi9@gmail.com
